# Supplementary material for: EFN-4/Ephrin converges with SAX-3/Robo, UNC-6/Netrin, and Heparan Sulfate Proteoglycan signaling to control MAB-5/Hox-dependent posterior Q neuroblast migration in Caenorhabditis elegans
Source: PLoS Genet. 2026 Sep 8;22(9):e1012303. doi: 10.1371/journal.pgen.1012303 (PMC13581211; doi:10.1371/journal.pgen.1012303)
Supplement: Supplementary file 1 [file pgen.1012303.s002.docx]

**Reviewers' comments:**

Reviewer's Responses to Questions

**Comments to the Authors:
Please note here if the review is uploaded as an attachment.**

Reviewer #1: Significance: How extracellular signals are transduced to affect cell polarity and migration movements is a major question in developmental cell biology. This paper delves into this important question using powerful classical genetics and smart deployment of cutting-edge technology like single cell transcriptomics and Alphafold3 predictions. The paper also synthesizes lines of evidence from previous -omics-scale work to make specific new insights that are then tested at a developmental genetic level. The experimental rigor is high, and the conclusions are well-supported by the results.

I have a question about the cell-autonomous vs. non-cell-autonomous role of UNC-5 in QL migration. The model presented in the Discussion depends on the relatively small fraction of UNC-5-expressing QL.a lineage cells shown in Fig. 3 and the lower (but nonzero) incidence of paradigm 2 defects in unc-5(ev480) compared to unc-5(e53). Could you expand upon the model a bit and clarify whether you are proposing that Netrin pathway activity via UNC-5 in a separate cell is required for proper PQR migration, or if the extracellular domain of UNC-5 itself is participating in this extracellular complex necessary for protrusion formation in QL?

As the reviewer observes, we were intrigued to see an effect of *unc-5* on QL.a migration but little to no expression of *unc-5* in the lineage, similar to *unc-6.* This suggests a non-autonomous role of UNC-5. That the *unc-5(ev480)* mutation specifically affects the cytoplasmic domain isoform of *unc-5* yet has minimal effects is consistent with a non-autonomous role of the UNC-5 extracellular domains in QL.a migration. The weak effect of *unc-5(ev480)* could be due to the fact that it introduces a premature stop codon which might lead to nonsense-mediated degradation of the long *unc-5* transcript, which might reduce overall UNC-5 levels resulting in a weak phenotype.

As we discussed on p. 26 in the Discussion, we propose that UNC-5, via its extracellular domain, forms a complex with UNC-6/Netrin and UNC-40/DCC and participates in the formation of the large extracellular complex. However, it is also possible that UNC-5 cytoplasmic signaling in a neighboring cell is required for QL.a migration. We moved this section to the Discussion of netrin signaling and added some details.

Additionally, in the table, when an entry has an * denoting a statistically significant difference, it is not always clear to what that entry is being compared.

The reviewer is correct that direct statistical comparisons are not included in the tables. Doing so made the tables extraordinarily complex and difficult to read. Therefore, we chose to describe the significant differences in the text as indicated with footnotes in the tables. If this is not sufficient, we will consider other options such as addenda below the tables describing the statistical comparisons.

My feedback has to do with the presentation of the Results text. Genetics work is dense, and the tabular presentation of most results in this paper is ideal. I would encourage selective descriptions in the Results text that focus on the rationale behind experiments, the key outcomes, and salient conclusions over restating of the numerical data that is in the tables. Such data is difficult to read in paragraph form and is already give succinctly in the table. The Results sections can add value by given context to the experiments and synthesizing the results. Some of the existing results statements summarize the findings in a very conservative manner that is weaker than the data could support, and I have pointed out some of these below. Stating the conclusions more forcefully would help propel the throughline of the argument. I found the parts of the results most helpful that were written in this mode:
While some genetic redundancies were found (e.g. in slt-1(eh15); unc-40(n324)),
these data suggest a previously unreported convergent role of the classical axon
guidance systems involving SAX-3/Robo and UNC-6/Netrin in posterior QL.a and QL.ap
migration. Genetic interactions broadly reveal that these pathways act in a similar
genetic pathway with EFN-4/Ephrin and MAB-20/Semaphorin. Possibly, each of these
signaling molecules converges to control the third and final step in QL.ap posterior
migration.

We attempted to balance accurate statistical representation with clarity of presentation. As mentioned above, we chose to discuss statistical comparisons in the text rather than represent the comparisons in the tables (for clarity). We choose to state our conclusions in a relatively conservative manner.

Terminology critique:
I know this was established in a 2025 study, but I find “paradigm 2” meaning “third stage defect” phrasing to be confusing. This is especially true when the tables present defects as cell positions in the worm that are labeled like this in the table footnotes:
3Position 4, paradigm 1 as described in Results.
4Position 4, paradigm 2 as described in Results.

So a cell can be in Position 4, which reflects a second stage paradigm 1 defect, or it can be in Position 4, which reflects a third stage paradigm 2 defect. This is a lot of non-concordant number terms. However, I understand if the preference is to keep terms as established in the original “paradigm 2” study. (I also want to flag here that the difference between 4.1 and 4.2 is not described in the Results as the footnote says, but is described in the Methods.) It would also be helpful to map Positions 1-5 on the schematics in Fig. 1.

As described in Jain and Lundquist 2025 and Figure 1C, the phases of migration represent the three migrations of QL.a and QL.ap, whereas the paradigms represent the placement of the PQR neuron in older animals (with paradigm 1 representing phase 1 and 2 failures and paradigm 2 representing phase 3 failure. We will indicate in Tables 1 and 4 that the 4.1 and 4.2 are described in the Materials and Methods and not the Results.

Places where the conclusions seem understated:
Given the wealth of information we have on these genes and their products, this seems like too understated of a conclusion:

Neither unc-6; unc-40 nor unc-5; unc-40 double mutants displayed significantly enhanced defects(Table 1), suggesting that they might act in the same pathway.

This conclusion is the strict interpretation of the genetic results and is consistent with the way our genetic data was interpreted throughout (described in the Materials and Methods section). However, given the wealth of what is known about these pathways, we add a statement saying this this is consistent with all of the previous data.

Likewise, this seems like a very conservative statement of the rescues with the Q lineage overexpression of mab-5 and efn-4 in the Slit/Robo and Netrin pathway mutants:
“These results are consistent with these molecules acting in a common
pathway to control posterior PQR migration wherein transgenic expression of one
molecule can partially compensate for loss of another.”

Again, we did try to be consistent about how we interpret genetic results throughout, which we agree can sound on the conservative side.

Places where the text is gene-/number-heavy and lacks context and examples of how context helps readability:

The genetics are complicated and their descriptions are necessarily verbose (especially when balancers and maternal rescue is involved). Therefore, this statement was very helpful, and could even appear in the introductory paragraph of the section, to set up what comes next:

In sum, these genetic interactions (a lack of strong phenotypic synergy) between
efn-4, mab-20, sax-3, slt-1, and unc-34 are consistent with the idea that these genes act
in a common pathway in posterior QL.a and QL.ap migration, with the exception being
potential parallel roles of the long isoform of SAX-3 and EFN-4 in the first two stages of
QL.a and QL.ap migration.

We agree that this statement would be useful as a preamble to the data that comes next. It is now at the beginning of the description of the results.

In an instance of a picture being worth a thousand words, this section associated with the compelling Fig.5 could use some context (even directing the reader to the Methods) for the presentation of the numerical scores from AlphaFold3:
Using the ECIA results from (NAWROCKA et al. 2024) as guidance, AlphaFold3
was used to model these potential interfacing domains. The D2 domain of EFN-4 was
modeled with various combinations of SAX-3 Ig domains. Ig domains 3 and 4 were
found to form the strongest interface, with an Interface Predicted Template Modeling
(ipTM) score of 0.82 and a Predicted Template Modeling (pTM) score of 0.61 (Figure
5A). The RBD domain of EFN-4 and Sema domain of MAB-20 were found to interface
strongly with an ipTM score of 0.74 and a pTM score of 0.7 (Figure 5B). For SAX-3 and
SLT-1, the strongest potential interaction was between the SAX-3 Ig1 domain and the
LRRNT2 domain of SLT-1, with an ipTM score of 0.66 and a pTM score of 0.63 (Figure
5C). This was achieved by folding the Ig1-2 domains of SAX-3 with the LRRNT1-3
domains of SLT-1. In the course of modeling interactions between these molecules, we
found a strong predicted dimerized fold of two copies of MAB-20, which exhibited an
ipTM score of 0.71 and a pTM score of 0.78 (Figure 5E).

A statement was added to clarify context behind numerical scores, along with citations
(Pei et al. 2026 <https://www.nature.com/articles/s41598-026-54567-0>)
(Merchant et al. 2025 https://www.nature.com/articles/s41586-025-09749-7)
(Wee and Wei 2024 https://pubs.acs.org/doi/10.1021/acs.jcim.4c00976).

It will help to evaluate a statement like this one:
This final fold yielded a modest ipTM score of 0.27 and a pTM score of 0.35. However, when
folding large protein structures with multiple interfaces and vast non-interfacing regions,
these scores can be considered to be reasonable indications that these select
interactions could possibly occur simultaneously.

We altered the text to read:

These low scores give low confidence in this prediction of a potential multimeric complex. However, analyses such as this can be confounded by large protein structures with multiple interfaces and vast non-interfacing regions. In any case, modeling with AlphaFold3 is consistent with the discrete physical interactions described in (Nawrocka et al., 2026).

This section just jumps in with results without any setup, which would be helpful to provide.
Genetic interactions between signaling pathways in posterior PQR migration
unc-6(ev400); sax-3(zy5) were sterile and were maintained as sax-3
heterozygotes. sax-3(zy5)M+; unc-6(ev400) double mutants did not differ significantly
from the predicted additive effect (Table 1).

Agreed. We add a statement justifying the thinking behind the experiments.

Could this (below) belong in a supplement instead of main body? Same could go for the sax-3(yad175) section. They feel like a diversion that muddies the water instead of clarifying.

This section:
The unc-6(e78) mutant, which more strongly affects dorsal than ventral axon guidance, displayed 50%, significantly more than unc-6(ev400) (p = 0.0013). These data indicate that UNC6/Netrin is involved in posterior QL.a migration, and that unc-6(e78) might not be a
simple loss-of-function.

We think these are interesting results for afficionados who might want to pursue these specific effects of *unc-6* and *sax-3* and therefore prefer to keep them in the Results.

DISCUSSION:
“Modeling with AlphaFold3 here supports the physical interactions
between EFN-4, MAB-20, SAX-3, and SLT-1 described in…”
I suggest rephrasing; the physical interaction would support a model predicting interaction, not the other way around. Perhaps phrase as “Modelling with AlphaFold3 predicts the nature/structure of physical interactions between EFN-4, MAB-20, SAX-3, and SLT-1 described in…”

We have reworded the section to reflect this comment.

In general, referencing of the ECIA interactome study could use a little more context upon its first introduction, including defining “connected community”. It had been a little while since I looked at it, and I had to remind myself of the details.

Agreed. We now discuss more details about the Nawrocka ECIA methods and conclusions.

The paper does an excellent job citing relevant worm literature, but the broader context could be improved. I appreciated the setup in the Introduction that ephrins are known to facilitate bidirectional signaling in other organisms. For example, that dimerized MAB-20 model is beautiful! Commentary on structural similarity of vertebrate semaphorins or if any other semaphorins are known to dimerize would be welcome.

The Sema domain of other Semaphorins is known to drive homodimerization. We now discuss and cite this in the text.

Typos/errors
In “lin-17 and sax-3 genetic interactions” section, third line, I think it should be “activates” not “activated”. In the last sentence of that section there is a redundant word: “prevent inhibit”

Done.

Fig. 6 legend is erroneously titled Fig. 5 on page 58

Done.

This transition seems out of order. It goes from setting up scoring PQR migration to gene expression results, then back to PQR defects:
As the paradigm 2 posterior PQR
migration defects were previously unappreciated, and the fact that sdn-1 affects PQR
migration, we scored PQR migration in viable HSPG mutants sdn-1/Syndecan, lon-
2/Glypican, gpn-1/Glypican, agr-1/Agrin, unc-52/Perlecan, and cle-1/CollagenXVIII.
sdn-1 was expressed robustly in the QL lineage, and lon-2 and agr-1 were not
(Figure 3)…
NEXT PARAGRAPH: As previously reported, sdn-1(zh20) and sdn-1(ok449) mutants displayed
posterior PQR migration defects (Table 4; Figure 7A).

Reworded. The expression section was moved to the end of the PQR scoring section.


Figure 2 legend: shouldn’t this say “paradigm 2”?
B) A sax-3(zy5) mutant animal with PQR
mispositioned immediately anterior to the anus (paradigm I PQR migration defect),

Done.

Reviewer #2: This study focuses on the migration of Q cell descendants in the developing C. elegans and characterizes several axon guidance pathway molecules to be involved in the process. Mainly through the use of loss-of-function mutations and testing for genetic interactions via double mutations, they reveal which proteins to be in the same or parallel pathways required for the migration of these cells, especially PQR. They conclude that EFN-4, MAB-20, SAX-3, SLT-1 (and the cytoskeletal protein unc-34/Ena) act in one pathway to direct the migration of QL descendants. The authors also show that heparin sulfate proteglycans, a syndecan (SDN-1) and a glypican (LON-1), and the Netrin pathway (UNC-5, UNC-6 and UNC-40) play roles, plausibly in parallel, for parts of the these migration events.

The manuscript is a dense and detailed description of numerous comparisons of phenotypes. There is little to dispute with the author’s interpretation of their phenotypic analysis and genetic interactions. However, it is not always clear within the tables which statistical comparisons are made without a deep dive in the text (esp. Table 3).

As mentioned in response to Reviewer 1, putting the exact statistical comparisons in the Tables made them too complex and difficult to discern. Therefore, we chose to explain the statistical tests in the text.

The authors also make predictions on a putative protein complex that might be mediating a hypothetical signal, to explain how SAX-3. SLT-1, MAB-20 and EFN-4 might be functioning in the same pathway. While these models may be possible and some quite plausible, they remain speculative. Specifically, the model in Fig. 5D is not supported by AlphaFold’s own confidence measures; the others are more defensible.

Agreed. The low scores give very low confidence in the prediction. We now state this in the text. The pairwise interactions have stronger scores and give insight into the possible nature of the physical interactions defined by the EICA. We prefer to keep in the statistical indicators.

Specific points:

1. Did the authors only use Fisher’s Exact text and no corrections of any sort? The description of the statistical tests is basic.

Fisher’s Exact tests were used for contingency (proportional) data. As most of these were pairwise comparisons, no correction for multiple testing was employed. We now discuss this in more detail in Materials and Methods.

2. Authors state that “when folding large protein structures with multiple interfaces and vast non-interfacing regions, these scores can be considered to be reasonable indications that these select interactions could possibly occur simultaneously”.

I am not aware of any conditions where an ipTM score of 0.27 and a pTM score of 0.35 are considered reasonable. The authors should justify this statement, cite supporting work, or replace it.

Agreed. We have now stated clearly that the low scores give very low confidence in the prediction of the multimeric complex.

3. Figure 5 (specifically panel D) is hard to interpret. It is not clear what the gray parts in the AlphaFold models represent. It is also not clear how biochemically plausible the model’s architecture is from the picture provided. For example, is the C-terminal end of EFN-4 accessible to a membrane for anchoring via a GPI linkage? Does the model agree with an architecture where EFN-4 and SAX-3 are on the same cell? It is hard to see this, but did the authors include signal peptides in their predicted proteins (which are removed from mature proteins).

The gray regions are now addressed in figure caption. They are predicted non-interfacing regions that were included in the folds, and colored regions are the predicted interfacial regions.

Signal peptides were not included in any predictions.

AlphaFold3 cannot predict if EFN-4 and SAX-3 can interact in *cis* in the same cell. We only analyze potential interactions of the indicated domains. While very interesting, we have not analyzed any potential steric effects of GPI linkage or protein crowding/bending on the plausibility of interactions in a complex.

4. The authors do not explain how AlphaFold predictions were made. If they did use Alphafold 3, did they use alphafoldserver.com from Google Deepmind? Did they use a local installation of another version (which version, github link?), or a Google Colab version? This questions arose since they claim to use Alphafold 3 for complex predictions, but then cite the manuscript for the AlphaFold Database (Varadi et al.), not the AlphaFold 3 paper (Abramson et al.) It may also help to include exact sequence boundaries/sequences used in AlphaFold predictions.

Abramson et al. 2024 is now referenced throughout, which was the basis for these analyses. We used the publically-available AlphaFold3 server <https://alphafoldserver.com>.

Sequence boundaries are present in materials and methods.

Materials and Methods has been modified to reflect these.

5. What is LRRNT1-3? Is that the N-terminal cap of an LRR domain? The three N-terminal LRR domains? The LRR N-terminal cap followed by the first three Leucine-rich repeats.

These are individual leucine rich repeats of SLT-1 which were included in the fold. LRRNT2 showed the interaction. We have reworded the manuscript to reflect this, and changed it in Figure 5.

6. The manuscript has a very long Discussion section that repeats much of the findings in the results. Authors are advised to avoid repetitions, to make the manuscript more manageable to read and digest.

We think it is important to re-state results and conclusions in the context of the Discussion where additional context is provided.

Reviewer #3: Leveraging a subtle but robust previously-undescribed phenotype, this work probes the implication of candidate genes selected from their previous results on single-cell transcriptomics on the QL lineage (the group has published and used single-cell transcriptomics on the QL lineage in two previous publications). The authors perform thorough phenotypic analyses of single and combinations of mutants, and cleverly integrate published information on physical interactions by Nawrocka et al., which offers elegant support and organizes the interpretation of complex genetic interactions.
This rigorous and well carried out work implicates known neurodevelopmental genes in the regulation of posterior neuroblast QL descendants’ migration. In particular, a novel phenotype characterized by defects in the third stage of posterior migration for the Q lineage helped to identify that SAX-3/Robo, UNC-6/Netrin, members of their signaling pathways, and heparan sulfate proteoglycans participate as regulators of this process. The genetic evidence presented is consistent with these factors converging onto a common pathway regulating posterior migration. Careful writing of genetic interpretations and excellent combination of published physical interactions results and predictive modelling to shed light onto potential multimeric molecular interactions. Predictive modeling analyses further indicate potential physical interactions among some of these components, but the modeling of the simultaneous interaction is weak and less convincing. Overall, this study provides new insights into the molecular mechanisms that regulate neuronal positioning during development.

Major concerns:

a. Lack of controls for recessivity of mutations for genes whose phenotypic effects needed to be tested in F1 progeny of heterozygous mothers (M+), as combining mutations in some double mutants were subviable. This alternative is much appreciated, but the recessivity of the mutations unc-6 (both alleles), unc-40, sax-3, slt-1, mab-20, and sdn-1 has never been explored before for this phenotype, and therefore these controls are required for enabling the genetic interpretations presented in the manuscript.

We note the examples where the mutants analyzed have wild-type maternal contribution due to sterility or sub-viability. The caveat is that the maternal contribution could be masking a phenotypic effect. We chose to analyze only homozygous interactions here. Potential haploinsufficiency could bolster our genetic arguments, but we did not explore potential haploinsufficiency here.

b. Need for detailed explanation and addition of references that are lacking to support the interpretation of the multimeric simultaneous interactions in the Alphafold predictive analyses. Better explain why the authors conclude that a simultaneous complex involving SAX-3, EFN-4, MAB-20 and SLT-1 (fig5d) might exist, as also schematized in final model (fig8). An ipTM value of 0.27 is quite low, which may be the case with larger complexes, but references and careful explanation needed.

Reviewer 2 had the same comment and concern, and we agree. The low scores indicate very low confidence in the prediction on the multimeric complex. We now state this in the text.

c. The authors use cell-specific Cas9 genome editing for testing sax-3 function in QL lineages. While this is a powerful approach, the tightness of egl-17 expression in QL is questionable, as egl-17 is also expressed in other cell types during embryogenesis and early larval development, including in rectal gland located near the migrating cell, based on transcriptomic data. As it stands, it is unclear that the gene loss of function defect arise from loss in QL specifically. The authors do mention 'Alternatively, sax-3 might also have non-autonomous effects on PQR migration', but this the broader expression of egl-17 should be explicitly stated and referenced.

The reviewer is correct that *egl-17* is expressed in tissues outside of the Q lineage, including the P epidermoblasts. However, in our hands, this expression occurs after Q lineage migration is complete. At the time of Q migration, *egl-17* expression in the posterior region is restricted to the Q lineages. We now state this in the text. We cannot rule out low levels of expression in other tissues and so state this in the text.

d. For sax-3, page 12 of pdf (Possibly, the long isoform is required only for the third and final QL.ap migration similar to efn-4, whereas the short isoform might be involved in the first, second, and/or third migrations): the authors rely on differential alleles to conclude about sax-3 isoforms. There seems to be a missed opportunity for directly testing the involvement of specific isoforms by using straightforward rescue assays with cDNAs for the different isoforms containing or not Ig domains 1-4.

Agreed. These experiments are planned for future studies. The focus of this work is genetic evidence for a large extracellular complex seeded by EFN-4 to control posterior migration.

e. The authors state 'lad-2 and sax-7 mutants had weak paradigm 2 PQR migration defects (2-7%), suggesting that might be involved in posterior QL.ap migration (Table 1). The role of lad-2 and sax-7 in posterior PQR migration will be the subject of future studies.' Since LAD-2 and LAD-1/SAX-7 are thought to function as scaffolding molecules, coordinating multiple interactions, it would very much strengthen the manuscript to include analysis of these genes in the current study as the final conclusion is the role of a multimeric complex.

We agree, but in this work we wanted to focus on genetic evidence for a large extracellular complex seeded by EFN-4. Detailed mechanistic analyses of distinct members of this complex are planned for future studies.

Minor concerns:

It would have been helpful to include page numbers and lines along the text for efficient referencing to issues.

Done.

1. A schematic representation of the classical guidance cues and its receptors would make the content easier to follow for a wider audience.

These classical pathways are described in the text. Figure 8 also shows the molecules.

2. A brief description of the roles of vab-8/KIF26, lin-17/Frizzled, and efn-4/Ephrin in migration signaling in the Introduction or earlier in the Results would improve readers’ understanding of the molecular context of the study.

The roles of these molecules are described in the first paragraph of the results section.

3. It would be helpful if the text explicitly stated the criteria used to identify the third stage, failed-migrating neuron as PQR rather than QLap, since it is not obvious whether the QLap cells that fail to migrate in paradigm 2 mutants are still able to differentiate into PQR. In line with this, it is not described if Pgcy-23::CFP is a marker for some or all of the QL descendants.

In Jain and Lundquist, 2025, we showed that QL.ap that failed in migration at stage 2 or 3 prematurely differentiated into PQR, as evidenced by extending a posterior dendritic protrusion characteristic of PQR, and exhibiting *gcy-23::cfp* expression. At the stage when PQR position was scored, QL.ap had already differentiated into PQR despite failed migration. We now mention this in the text.

4. Results: The stepwise posterior migration of QL.a and QL.ap defines a new PQR migration phenotype, page 10. PQR misplacement immediately posterior to the anus was defined as paradigm 2 PQR migration failure at the third and final step... It should say anterior instead of posterior.

Done.

5. Figure 2 (legend), page 50, panel B: A sax-3(zy5) mutant animal with PQR mispositioned immediately anterior to the anus (paradigm I PQR migration defect), indicative of a failure in the third and final QL.ap migration. Shouldn't it say paradigm 2?

Done.

6. The choice of the word 'paradigm' is unusual and less clear than 'phenotypic class' or 'defect category' or just 'class'?

This term was used in Jain and Lundquist 2025.

7. Computational prediction of a multi-component interaction between EFN-4, SAX-3, SLT-1 and MAB-20, page 19: However, when folding large protein structures with multiple interfaces and vast non-interfacing regions, these scores can be considered to be reasonable indications that these select interactions could possibly occur simultaneously. Include references.

Other reviewers noted that the low scores give very low confidence in the prediction. We now state this in the text and remove this statement.

8. remind authors of the nature of alleles sax-3(zy5 and zy152)

This is described in Results and details given in Materials and Methods.

9. Material and Methods: Scoring AQR and PQR locations, page 32. To score the location of both AQR and PQR neurons, we utilized the lqIs58 and lqIs244 [Pgcy-32::cfp]. The last bracket is missing.

Done.

10. Material and Methods: Statistical modeling of genetic interactions, page 32. Explaining the rationale for applying Fisher’s Exact test to assess the significance of proportional difference between genotypes in this study would help, as the choice for Fisher's Exact test is not justified.

Fisher’s exact test is a contingency test commonly used to analyze proportional data, including our past work with AQR and PQR migration. The alternative would be a Chi square test. Fisher’s test is more accurate because it is an “exact” calculation of difference and does not rely on normal distribution estimates as does Chi square.

11. Discussion: An autocrine reverse signaling mechanism of EFN-4/Ephrin, page 30. This is, in essence, reverse signaling via EFN-4/Ephrin. References need to be included. For instance, Holland et al., 1996; Bruckner et al., 1997; Kullander and Klein, 2002.

Done

12. References issues:
Nawrocka et al has been published in Cell Genomics in 2026.
Update citation from 2024 bioRxiv.
Texeira two citations bioRxiv and PLoS One paper, leave only journal publication

Done.

13. Many spelling mistakes:
Figure 1 (caption), 48, line 6: plase of birth (place of birth).
Fig 1: wild-type no hyphen , no italics
Space missing between 10 and uM in several Fig legends
Figure legend 5 should say '6'.
Figure legend 7. lon-1 --> lon-2
Fig 8 'UNC-34' on one line for clarity of reading
Spelling of EICA -> ECIA, first appearance.
lqIs221 Pegl-15 should be Pegl-17
page 8 of pdf, delete 'The' in last line 'The mutants....'
Word missing in interpretation sentence about lad-2 and sax-7
vab-8 was expressed predominantly in the QLa : dot missing .a
prevent inhibit: 'normally prevent inhibit activation of mab-5 in QL revealed in the lin- 17 background.'

All corrected.
