# Supplementary material for: EFN-4/Ephrin converges with SAX-3/Robo, UNC-6/Netrin, and Heparan Sulfate Proteoglycan signaling to control MAB-5/Hox-dependent posterior Q neuroblast migration in Caenorhabditis elegans
Source: PLoS Genet. 2026 Sep 8;22(9):e1012303. doi: 10.1371/journal.pgen.1012303 (PMC13581211; doi:10.1371/journal.pgen.1012303)
Supplement: Supplementary file 2 [file pgen.1012303.s003.docx]

Reviewer #1: Other than catching some typos, most of my initial suggestions pertained to data presentation. The authors added additional context where requested, and this improved readability of the paper. Certain stylistic choices were retained, which of course is the authors' right. I have one further minor suggestion, which is to specify the organism in the sentence with the single mention of SEMA4D.

Human. Done.

Reviewer #2: While the manuscript remains difficult to parse through for anyone who is not an expert on these genes and the existing C. elegans genetics literature, the stylistic choices are for the authors to make. Regardless, the revised manuscript is improved.

Minor points:

* "the strongest potential interaction was between the SAX-3 Ig1 domain and the second leucine-rich repeat (LRRNT2) domain of SLT-1"

LRRNT usually means LRR domain N-terminal region or cap (they can conform this via a google or AI search). Nearly every extracellular LRR domain contains one N-terminal (NT) and one C-terminal (CT) cap, usually rich in cysteines, to tie the loose ends of the leucine-rich repeats. By "LRRNT2", the authors mean the leucine-rich repeat domain 2 (all Slits have four LRR domains, so this is the second), and not the NT region/cap within this domain. I would remove the "NT" part, and use LRR Domain 2, LRR D2, LRR 2 or something similar.

We now refer to this region as LRR2. We changed this in Figure 5 and in the text.

* While the issue with low ipTM score for the "four-way" multimer remains, the authors have significantly improved their description and report the model as "low confidence". A less objectionable way could be to manually assemble their high-confidence binary complexes into a large complex rather than reporting a low-confidence four-way multimer, but the outcome would likely be very similar, so it's not worth it.

* As it is difficult to interpret and use the multimeric model the authors report in Fig. 5D, the authors/journal should provide the model (and the other AF models) as a supplemental data file in the form of coordinates (e.g., .pdb or .cif files), which would be ≤1 MB each. Otherwise, it is nearly impossible to test and confirm (or invalidate) their models via site-directed mutagenesis of the proposed interface.

We have deposited the models in ModelArchive as requested. A link I included in the manuscript (https://modelarchive.org/doi/10.5452/ma-lund-qmig).

Reviewer #3: The authors have satisfactorily addressed most issues raised, including adding precisions and comments in the text for genetic interactions and AlphaFold predictions, correcting reference citations, and having added Table footnotes for statistical analysis of comparisons. The text flow is much improved.
However, providing exact P-Values for Tables comparisons should be included in an addendum or simple Excel file containing all statistical comparisons so that it is clear what comparisons are being made, as offered by the authors. Key results could be bolded on Excel files.

We have added a column to Tables 1, 3, and 4 showing the Exact *p* values and the relevant comparison for significant differences. In Table 2, all comparisons are to *Pegl-17::mab-5* alone, which is now stated in the footnotes.

There are typos in new written sections. For example:
- l. 862 states sax-3(ky152), when it should be zy152
- In Abstract, l.62, 'the’ novel idea (not ‘that’)

Done.
