# Supplementary material for: EFN-4/Ephrin converges with SAX-3/Robo, UNC-6/Netrin, and Heparan Sulfate Proteoglycan signaling to control MAB-5/Hox-dependent posterior Q neuroblast migration in Caenorhabditis elegans
Source: PLoS Genet. 2026 Sep 8;22(9):e1012303. doi: 10.1371/journal.pgen.1012303 (PMC13581211; doi:10.1371/journal.pgen.1012303)
Supplement: Peer review history

# Peer review: Decision Letter 0

**DOI:** 10.1371/journal.pgen.1012303.r001

**Authors:** Giovanni Bosco, Jeffrey Essner

4 Jun 2026

PGENETICS-D-26-00323

EFN-4/Ephrin converges with SAX-3/Robo, UNC-6/Netrin, and Heparan Sulfate Proteoglycan signaling to control MAB-5/Hox-dependent posterior Q neuroblast migration in Caenorhabditis elegans

PLOS Genetics

Dear Dr. Lundquist,

Thank you for submitting your manuscript to PLOS Genetics. After careful consideration, we feel that it has merit. The reviewers have suggested many points to improve the manuscript and its readability. Therefore, we invite you to submit a revised version of the manuscript that addresses the points raised during the review process.

Please submit your revised manuscript within by Jul 04 2026 11:59PM. If you will need more time than this to complete your revisions, please reply to this message or contact the journal office at plosgenetics@plos.org. When you're ready to submit your revision, log on to https://www.editorialmanager.com/pgenetics/ and select the 'Submissions Needing Revision' folder to locate your manuscript file.

Please include the following items when submitting your revised manuscript:

* A letter that responds to each point raised by the editor and reviewer(s). You should upload this letter as a separate file labeled 'Response to Reviewers'. This file does not need to include responses to formatting updates and technical items listed in the 'Journal Requirements' section below.

* A marked-up copy of your manuscript that highlights changes made to the original version. You should upload this as a separate file labeled 'Revised Manuscript with Track Changes'.

* An unmarked version of your revised paper without tracked changes. You should upload this as a separate file labeled 'Manuscript'.

If you would like to make changes to your financial disclosure, competing interests statement, or data availability statement, please make these updates within the submission form at the time of resubmission. Guidelines for resubmitting your figure files are available below the reviewer comments at the end of this letter.

As the corresponding author, your ORCID iD is verified in the submission system and will appear in the published article. PLOS supports the use of ORCID, and we encourage all coauthors to register for an ORCID iD and use it as well. Please encourage your coauthors to verify their ORCID iD within the submission system before final acceptance, as unverified ORCID iDs will not appear in the published article. Only the individual author can complete the verification step; PLOS staff cannot verify ORCID iDs on behalf of authors.

We look forward to receiving your revised manuscript.

Kind regards,

Jeffrey J Essner

Academic Editor

PLOS Genetics

Giovanni Bosco

Section Editor

PLOS Genetics

Aimée Dudley

Editor-in-Chief

PLOS Genetics

Anne Goriely

Editor-in-Chief

PLOS Genetics

**Journal Requirements:**

1) We ask that a manuscript source file is provided at Revision. Please upload your manuscript file as a .doc, .docx, .rtf or .tex. If you are providing a .tex file, please upload it under the item type u2018LaTeX Source Fileu2019 and leave your .pdf version as the item type u2018Manuscriptu2019.

2) Please provide an Author Summary. This should appear in your manuscript between the Abstract (if applicable) and the Introduction, and should be 150-200 words long. The aim should be to make your findings accessible to a wide audience that includes both scientists and non-scientists. Sample summaries can be found on our website under Submission Guidelines:

https://journals.plos.org/plosgenetics/s/submission-guidelines#loc-parts-of-a-submission

3) Please upload all main figures as separate Figure files in .tif or .eps format. For more information about how to convert and format your figure files please see our guidelines:

https://journals.plos.org/plosgenetics/s/figures

**Reviewers' comments:**

Reviewer's Responses to Questions

**Comments to the Authors:**

**Please note here if the review is uploaded as an attachment.**

Reviewer #1: Significance: How extracellular signals are transduced to affect cell polarity and migration movements is a major question in developmental cell biology. This paper delves into this important question using powerful classical genetics and smart deployment of cutting-edge technology like single cell transcriptomics and Alphafold3 predictions. The paper also synthesizes lines of evidence from previous -omics-scale work to make specific new insights that are then tested at a developmental genetic level. The experimental rigor is high, and the conclusions are well-supported by the results.

I have a question about the cell-autonomous vs. non-cell-autonomous role of UNC-5 in QL migration. The model presented in the Discussion depends on the relatively small fraction of UNC-5-expressing QL.a lineage cells shown in Fig. 3 and the lower (but nonzero) incidence of paradigm 2 defects in unc-5(ev480) compared to unc-5(e53). Could you expand upon the model a bit and clarify whether you are proposing that Netrin pathway activity via UNC-5 in a separate cell is required for proper PQR migration, or if the extracellular domain of UNC-5 itself is participating in this extracellular complex necessary for protrusion formation in QL?

Additionally, in the table, when an entry has an * denoting a statistically significant difference, it is not always clear to what that entry is being compared.

My feedback has to do with the presentation of the Results text. Genetics work is dense, and the tabular presentation of most results in this paper is ideal. I would encourage selective descriptions in the Results text that focus on the rationale behind experiments, the key outcomes, and salient conclusions over restating of the numerical data that is in the tables. Such data is difficult to read in paragraph form and is already give succinctly in the table. The Results sections can add value by given context to the experiments and synthesizing the results. Some of the existing results statements summarize the findings in a very conservative manner that is weaker than the data could support, and I have pointed out some of these below. Stating the conclusions more forcefully would help propel the throughline of the argument.

I found the parts of the results most helpful that were written in this mode:

While some genetic redundancies were found (e.g. in slt-1(eh15); unc-40(n324)),

these data suggest a previously unreported convergent role of the classical axon

guidance systems involving SAX-3/Robo and UNC-6/Netrin in posterior QL.a and QL.ap

migration. Genetic interactions broadly reveal that these pathways act in a similar

genetic pathway with EFN-4/Ephrin and MAB-20/Semaphorin. Possibly, each of these

signaling molecules converges to control the third and final step in QL.ap posterior

migration.

Terminology critique:

I know this was established in a 2025 study, but I find “paradigm 2” meaning “third stage defect” phrasing to be confusing. This is especially true when the tables present defects as cell positions in the worm that are labeled like this in the table footnotes:

3Position 4, paradigm 1 as described in Results.

4Position 4, paradigm 2 as described in Results.

So a cell can be in Position 4, which reflects a second stage paradigm 1 defect, or it can be in Position 4, which reflects a third stage paradigm 2 defect. This is a lot of non-concordant number terms. However, I understand if the preference is to keep terms as established in the original “paradigm 2” study. (I also want to flag here that the difference between 4.1 and 4.2 is not described in the Results as the footnote says, but is described in the Methods.) It would also be helpful to map Positions 1-5 on the schematics in Fig. 1.

Places where the conclusions seem understated:

Given the wealth of information we have on these genes and their products, this seems like too understated of a conclusion:

Neither unc-6; unc-40 nor unc-5; unc-40 double mutants displayed significantly enhanced defects(Table 1), suggesting that they might act in the same pathway.

Likewise, this seems like a very conservative statement of the rescues with the Q lineage overexpression of mab-5 and efn-4 in the Slit/Robo and Netrin pathway mutants:

“These results are consistent with these molecules acting in a common

pathway to control posterior PQR migration wherein transgenic expression of one

molecule can partially compensate for loss of another.”

Places where the text is gene-/number-heavy and lacks context and examples of how context helps readability:

The genetics are complicated and their descriptions are necessarily verbose (especially when balancers and maternal rescue is involved). Therefore, this statement was very helpful, and could even appear in the introductory paragraph of the section, to set up what comes next:

In sum, these genetic interactions (a lack of strong phenotypic synergy) between

efn-4, mab-20, sax-3, slt-1, and unc-34 are consistent with the idea that these genes act

in a common pathway in posterior QL.a and QL.ap migration, with the exception being

potential parallel roles of the long isoform of SAX-3 and EFN-4 in the first two stages of

QL.a and QL.ap migration.

In an instance of a picture being worth a thousand words, this section associated with the compelling Fig.5 could use some context (even directing the reader to the Methods) for the presentation of the numerical scores from AlphaFold3:

Using the ECIA results from (NAWROCKA et al. 2024) as guidance, AlphaFold3

was used to model these potential interfacing domains. The D2 domain of EFN-4 was

modeled with various combinations of SAX-3 Ig domains. Ig domains 3 and 4 were

found to form the strongest interface, with an Interface Predicted Template Modeling

(ipTM) score of 0.82 and a Predicted Template Modeling (pTM) score of 0.61 (Figure

5A). The RBD domain of EFN-4 and Sema domain of MAB-20 were found to interface

strongly with an ipTM score of 0.74 and a pTM score of 0.7 (Figure 5B). For SAX-3 and

SLT-1, the strongest potential interaction was between the SAX-3 Ig1 domain and the

LRRNT2 domain of SLT-1, with an ipTM score of 0.66 and a pTM score of 0.63 (Figure

5C). This was achieved by folding the Ig1-2 domains of SAX-3 with the LRRNT1-3

domains of SLT-1. In the course of modeling interactions between these molecules, we

found a strong predicted dimerized fold of two copies of MAB-20, which exhibited an

ipTM score of 0.71 and a pTM score of 0.78 (Figure 5E).

It will help to evaluate a statement like this one:

This final fold yielded a modest ipTM score of 0.27 and a pTM score of 0.35. However, when

folding large protein structures with multiple interfaces and vast non-interfacing regions,

these scores can be considered to be reasonable indications that these select

interactions could possibly occur simultaneously.

This section just jumps in with results without any setup, which would be helpful to provide.

Genetic interactions between signaling pathways in posterior PQR migration

unc-6(ev400); sax-3(zy5) were sterile and were maintained as sax-3

heterozygotes. sax-3(zy5)M+; unc-6(ev400) double mutants did not differ significantly

from the predicted additive effect (Table 1).

Could this (below) belong in a supplement instead of main body? Same could go for the sax-3(yad175) section. They feel like a diversion that muddies the water instead of clarifying.

This section:

The unc-6(e78) mutant, which more strongly affects dorsal than ventral axon guidance, displayed 50%, significantly more than unc-6(ev400) (p = 0.0013). These data indicate that UNC6/Netrin is involved in posterior QL.a migration, and that unc-6(e78) might not be a

simple loss-of-function.

DISCUSSION:

“Modeling with AlphaFold3 here supports the physical interactions

between EFN-4, MAB-20, SAX-3, and SLT-1 described in…”

I suggest rephrasing; the physical interaction would support a model predicting interaction, not the other way around. Perhaps phrase as “Modelling with AlphaFold3 predicts the nature/structure of physical interactions between EFN-4, MAB-20, SAX-3, and SLT-1 described in…”

In general, referencing of the ECIA interactome study could use a little more context upon its first introduction, including defining “connected community”. It had been a little while since I looked at it, and I had to remind myself of the details.

The paper does an excellent job citing relevant worm literature, but the broader context could be improved. I appreciated the setup in the Introduction that ephrins are known to facilitate bidirectional signaling in other organisms. For example, that dimerized MAB-20 model is beautiful! Commentary on structural similarity of vertebrate semphorins or if any other semaphorins are known to dimerize would be welcome.

Typos/errors

In “lin-17 and sax-3 genetic interactions” section, third line, I think it should be “activates” not “activated”. In the last sentence of that section there is a redundant word: “prevent inhibit”

Fig. 6 legend is erroneously titled Fig. 5 on page 58

This transition seems out of order. It goes from setting up scoring PQR migration to gene expression results, then back to PQR defects:

As the paradigm 2 posterior PQR

migration defects were previously unappreciated, and the fact that sdn-1 affects PQR

migration, we scored PQR migration in viable HSPG mutants sdn-1/Syndecan, lon-

2/Glypican, gpn-1/Glypican, agr-1/Agrin, unc-52/Perlecan, and cle-1/CollagenXVIII.

sdn-1 was expressed robustly in the QL lineage, and lon-2 and agr-1 were not

(Figure 3)…

NEXT PARAGRAPH: As previously reported, sdn-1(zh20) and sdn-1(ok449) mutants displayed

posterior PQR migration defects (Table 4; Figure 7A).

Figure 2 legend: shouldn’t this say “paradigm 2”?

B) A sax-3(zy5) mutant animal with PQR

mispositioned immediately anterior to the anus (paradigm I PQR migration defect),

Reviewer #2: This study focuses on the migration of Q cell descendants in the developing C. elegans and characterizes several axon guidance pathway molecules to be involved in the process. Mainly through the use of loss-of-function mutations and testing for genetic interactions via double mutations, they reveal which proteins to be in the same or parallel pathways required for the migration of these cells, especially PQR. They conclude that EFN-4, MAB-20, SAX-3, SLT-1 (and the cytoskeletal protein unc-34/Ena) act in one pathway to direct the migration of QL descendants. The authors also show that heparin sulfate proteglycans, a syndecan (SDN-1) and a glypican (LON-1), and the Netrin pathway (UNC-5, UNC-6 and UNC-40) play roles, plausibly in parallel, for parts of the these migration events.

The manuscript is a dense and detailed description of numerous comparisons of phenotypes. There is little to dispute with the author’s interpretation of their phenotypic analysis and genetic interactions. However, it is not always clear within the tables which statistical comparisons are made without a deep dive in the text (esp. Table 3).

The authors also make predictions on a putative protein complex that might be mediating a hypothetical signal, to explain how SAX-3. SLT-1, MAB-20 and EFN-4 might be functioning in the same pathway. While these models may be possible and some quite plausible, they remain speculative. Specifically, the model in Fig. 5D is not supported by AlphaFold’s own confidence measures; the others are more defensible.

Specific points:

1. Did the authors only use Fisher’s Exact text and no corrections of any sort? The description of the statistical tests is basic.

2. Authors state that “when folding large protein structures with multiple interfaces and vast non-interfacing regions, these scores can be considered to be reasonable indications that these select interactions could possibly occur simultaneously”.

I am not aware of any conditions where an ipTM score of 0.27 and a pTM score of 0.35 are considered reasonable. The authors should justify this statement, cite supporting work, or replace it.

3. Figure 5 (specifically panel D) is hard to interpret. It is not clear what the gray parts in the AlphaFold models represent. It is also not clear how biochemically plausible the model’s architecture is from the picture provided. For example, is the C-terminal end of EFN-4 accessible to a membrane for anchoring via a GPI linkage? Does the model agree with an architecture where EFN-4 and SAX-3 are on the same cell? It is hard to see this, but did the authors include signal peptides in their predicted proteins (which are removed from mature proteins).

4. The authors do not explain how AlphaFold predictions were made. If they did use Alphafold 3, did they use alphafoldserver.com from Google Deepmind? Did they use a local installation of another version (which version, github link?), or a Google Colab version? This questions arose since they claim to use Alphafold 3 for complex predictions, but then cite the manuscript for the AlphaFold Database (Varadi et al.), not the AlphaFold 3 paper (Abramson et al.) It may also help to include exact sequence boundaries/sequences used in AlphaFold predictions.

5. What is LRRNT1-3? Is that the N-terminal cap of an LRR domain? The three N-terminal LRR domains? The LRR N-terminal cap followed by the first three Leucine-rich repeats.

6. The manuscript has a very long Discussion section that repeats much of the findings in the results. Authors are advised to avoid repetitions, to make the manuscript more manageable to read and digest.

Reviewer #3: Leveraging a subtle but robust previously-undescribed phenotype, this work probes the implication of candidate genes selected from their previous results on single-cell transcriptomics on the QL lineage (the group has published and used single-cell transcriptomics on the QL lineage in two previous publications). The authors perform thorough phenotypic analyses of single and combinations of mutants, and cleverly integrate published information on physical interactions by Nawrocka et al., which offers elegant support and organizes the interpretation of complex genetic interactions.

This rigorous and well carried out work implicates known neurodevelopmental genes in the regulation of posterior neuroblast QL descendants’ migration. In particular, a novel phenotype characterized by defects in the third stage of posterior migration for the Q lineage helped to identify that SAX-3/Robo, UNC-6/Netrin, members of their signaling pathways, and heparan sulfate proteoglycans participate as regulators of this process. The genetic evidence presented is consistent with these factors converging onto a common pathway regulating posterior migration. Careful writing of genetic interpretations and excellent combination of published physical interactions results and predictive modelling to shed light onto potential multimeric molecular interactions. Predictive modeling analyses further indicate potential physical interactions among some of these components, but the modeling of the simultaneous interaction is weak and less convincing. Overall, this study provides new insights into the molecular mechanisms that regulate neuronal positioning during development.

Major concerns:

a. Lack of controls for recessivity of mutations for genes whose phenotypic effects needed to be tested in F1 progeny of heterozygous mothers (M+), as combining mutations in some double mutants were subviable. This alternative is much appreciated, but the recessivity of the mutations unc-6 (both alleles), unc-40, sax-3, slt-1, mab-20, and sdn-1 has never been explored before for this phenotype, and therefore these controls are required for enabling the genetic interpretations presented in the manuscript.

b. Need for detailed explanation and addition of references that are lacking to support the interpretation of the multimeric simultaneous interactions in the Alphafold predictive analyses. Better explain why the authors conclude that a simultaneous complex involving SAX-3, EFN-4, MAB-20 and SLT-1 (fig5d) might exist, as also schematized in final model (fig8). An ipTM value of 0.27 is quite low, which may be the case with larger complexes, but references and careful explanation needed.

c. The authors use cell-specific Cas9 genome editing for testing sax-3 function in QL lineages. While this is a powerful approach, the tightness of egl-17 expression in QL is questionable, as egl-17 is also expressed in other cell types during embryogenesis and early larval development, including in rectal gland located near the migrating cell, based on transcriptomic data. As it stands, it is unclear that the gene loss of function defect arise from loss in QL specifically. The authors do mention 'Alternatively, sax-3 might also have non-autonomous effects on PQR migration', but this the broader expression of egl-17 should be explicitly stated and referenced.

d. For sax-3, page 12 of pdf (Possibly, the long isoform is required only for the third and final QL.ap migration similar to efn-4, whereas the short isoform might be involved in the first, second, and/or third migrations): the authors rely on differential alleles to conclude about sax-3 isoforms. There seems to be a missed opportunity for directly testing the involvement of specific isoforms by using straightforward rescue assays with cDNAs for the different isoforms containing or not Ig domains 1-4.

e. The authors state 'lad-2 and sax-7 mutants had weak paradigm 2 PQR migration defects (2-7%), suggesting that might be involved in posterior QL.ap migration (Table 1). The role of lad-2 and sax-7 in posterior PQR migration will be the subject of future studies.' Since LAD-2 and LAD-1/SAX-7 are thought to function as scaffolding molecules, coordinating multiple interactions, it would very much strengthen the manuscript to include analysis of these genes in the current study as the final conclusion is the role of a multimeric complex.

Minor concerns:

It would have been helpful to include page numbers and lines along the text for efficient referencing to issues.

1. A schematic representation of the classical guidance cues and its receptors would make the content easier to follow for a wider audience.

2. A brief description of the roles of vab-8/KIF26, lin-17/Frizzled, and efn-4/Ephrin in migration signaling in the Introduction or earlier in the Results would improve readers’ understanding of the molecular context of the study.

3. It would be helpful if the text explicitly stated the criteria used to identify the third stage, failed-migrating neuron as PQR rather than QLap, since it is not obvious whether the QLap cells that fail to migrate in paradigm 2 mutants are still able to differentiate into PQR. In line with this, it is not described if Pgcy-23::CFP is a marker for some or all of the QL descendants.

4. Results: The stepwise posterior migration of QL.a and QL.ap defines a new PQR migration phenotype, page 10. PQR misplacement immediately posterior to the anus was defined as paradigm 2 PQR migration failure at the third and final step... It should say anterior instead of posterior.

5. Figure 2 (legend), page 50, panel B: A sax-3(zy5) mutant animal with PQR mispositioned immediately anterior to the anus (paradigm I PQR migration defect), indicative of a failure in the third and final QL.ap migration. Shouldn't it say paradigm 2?

6. The choice of the word 'paradigm' is unusual and less clear than 'phenotypic class' or 'defect category' or just 'class'?

7. Computational prediction of a multi-component interaction between EFN-4, SAX-3, SLT-1 and MAB-20, page 19: However, when folding large protein structures with multiple interfaces and vast non-interfacing regions, these scores can be considered to be reasonable indications that these select interactions could possibly occur simultaneously. Include references.

8. remind authors of nature of alleles sax-3(zy5 and zy152)

9. Material and Methods: Scoring AQR and PQR locations, page 32. To score the location of both AQR and PQR neurons, we utilized the lqIs58 and lqIs244 [Pgcy-32::cfp]. The last bracket is missing.

10. Material and Methods: Statistical modeling of genetic interactions, page 32. Explaining the rationale for applying Fisher’s Exact test to assess the significance of proportional difference between genotypes in this study would help, as the choice for Fisher's Exact test is not justified.

11. Discussion: An autocrine reverse signaling mechanism of EFN-4/Ephrin, page 30. This is, in essence, reverse signaling via EFN-4/Ephrin. References need to be included. For instance, Holland et al., 1996; Bruckner et al., 1997; Kullander and Klein, 2002.

12. References issues:

Nawrocka et al has been published in Cell Genomics in 2026.

Update citation from 2024 bioRxiv.

Texeira two citations bioRxiv and PLoS One paper, leave only journal publication

13. Many spelling mistakes:

Figure 1 (caption), 48, line 6: plase of birth (place of birth).

Fig 1: wild-type no hyphen , no italics

Space missing between 10 and uM in several Fig legends

Figure legend 5 should say '6'.

Figure legend 7. lon-1 --> lon-2

Fig 8 'UNC-34' on one line for clarity of reading

Spelling of EICA -> ECIA, first appearance.

lqIs221 Pegl-15 should be Pegl-17

page 8 of pdf, delete 'The' in last line 'The mutants....'

Word missing in interpretation sentence about lad-2 and sax-7

vab-8 was expressed predominantly in the QLa : dot missing .a

prevent inhibit: 'normally prevent inhibit activation of mab-5 in QL revealed in the lin- 17 background.'

**********

**Have all data underlying the figures and results presented in the manuscript been provided?**

Large-scale datasets should be made available via a public repository as described in the *PLOS Genetics*  data availability policy, and numerical data that underlies graphs or summary statistics should be provided in spreadsheet form as supporting information.

Reviewer #1: Yes

Reviewer #2: Yes

Reviewer #3: **No:** Statistical tests with exact p values not provided (perhaps they are provided in Source data, but such a file is not available in the PDF received to evaluate the manuscript). Hard to evaluate on Tables of penetrances alone.

**********

PLOS authors have the option to publish the peer review history of their article (what does this mean?). If published, this will include your full peer review and any attached files.

If you choose “no”, your identity will remain anonymous but your review may still be made public.

**Do you want your identity to be public for this peer review?**  For information about this choice, including consent withdrawal, please see our Privacy Policy.

Reviewer #1: No

Reviewer #2: No

Reviewer #3: No

[NOTE: If reviewer comments were submitted as an attachment file, they will be attached to this email and accessible via the submission site. Please log into your account, locate the manuscript record, and check for the action link "View Attachments". If this link does not appear, there are no attachment files.]

**Figure resubmission:**

**Reproducibility:**

To enhance the reproducibility of your results, we recommend that authors deposit laboratory protocols in protocols.io, where a protocol can be assigned its own identifier (DOI) such that it can be cited independently in the future. Additionally, PLOS ONE offers an option to publish peer-reviewed clinical study protocols. Read more information on sharing protocols at https://plos.org/protocols?utm_medium=editorial-email&utm_source=authorletters&utm_campaign=protocols

# Author comment: Author response to Decision Letter 1

**DOI:** 10.1371/journal.pgen.1012303.r002

1 Jul 2026

Attachment Submitted filename: Response to reviewer comments.docx

# Peer review: Decision Letter 1

**DOI:** 10.1371/journal.pgen.1012303.r003

**Authors:** Giovanni Bosco, Jeffrey Essner

4 Aug 2026

PGENETICS-D-26-00323R1

EFN-4/Ephrin converges with SAX-3/Robo, UNC-6/Netrin, and Heparan Sulfate Proteoglycan signaling to control MAB-5/Hox-dependent posterior Q neuroblast migration in Caenorhabditis elegans

PLOS Genetics

Dear Dr. Lundquist,

Thank you for submitting your manuscript to PLOS Genetics. After careful consideration, we feel that it has merit but does not fully meet PLOS Genetics's publication criteria as it currently stands. Therefore, we invite you to submit a revised version of the manuscript that addresses the points raised during the review process.

Please submit your revised manuscript within by Sep 03 2026 11:59PM. If you will need more time than this to complete your revisions, please reply to this message or contact the journal office at plosgenetics@plos.org. When you're ready to submit your revision, log on to https://www.editorialmanager.com/pgenetics/ and select the 'Submissions Needing Revision' folder to locate your manuscript file.

Please include the following items when submitting your revised manuscript:

* A letter that responds to each point raised by the editor and reviewer(s). You should upload this letter as a separate file labeled 'Response to Reviewers'. This file does not need to include responses to formatting updates and technical items listed in the 'Journal Requirements' section below.

* A marked-up copy of your manuscript that highlights changes made to the original version. You should upload this as a separate file labeled 'Revised Manuscript with Track Changes'.

* An unmarked version of your revised paper without tracked changes. You should upload this as a separate file labeled 'Manuscript'.

If you would like to make changes to your financial disclosure, competing interests statement, or data availability statement, please make these updates within the submission form at the time of resubmission. Guidelines for resubmitting your figure files are available below the reviewer comments at the end of this letter.

As the corresponding author, your ORCID iD is verified in the submission system and will appear in the published article. PLOS supports the use of ORCID, and we encourage all coauthors to register for an ORCID iD and use it as well. Please encourage your coauthors to verify their ORCID iD within the submission system before final acceptance, as unverified ORCID iDs will not appear in the published article. Only the individual author can complete the verification step; PLOS staff cannot verify ORCID iDs on behalf of authors.

We look forward to receiving your revised manuscript.

Kind regards,

Jeffrey J Essner

Academic Editor

PLOS Genetics

Giovanni Bosco

Section Editor

PLOS Genetics

Aimée Dudley

Editor-in-Chief

PLOS Genetics

Anne Goriely

Editor-in-Chief

PLOS Genetics

**Additional Editor Comments (if provided):**

Please see the reviewers comments and address these if you can. Most comments are minor.

**Reviewers' comments:**

Reviewer's Responses to Questions

**Comments to the Authors:**

**Please note here if the review is uploaded as an attachment.**

Reviewer #1: Other than catching some typos, most of my initial suggestions pertained to data presentation. The authors added additional context where requested, and this improved readability of the paper. Certain stylistic choices were retained, which of course is the authors' right. I have one further minor suggestion, which is to specify the organism in the sentence with the single mention of SEMA4D.

Reviewer #2: While the manuscript remains difficult to parse through for anyone who is not an expert on these genes and the existing C. elegans genetics literature, the stylistic choices are for the authors to make. Regardless, the revised manuscript is improved.

Minor points:

* "the strongest potential interaction was between the SAX-3 Ig1 domain and the second leucine-rich repeat (LRRNT2) domain of SLT-1"

LRRNT usually means LRR domain N-terminal region or cap (they can conform this via a google or AI search). Nearly every extracellular LRR domain contains one N-terminal (NT) and one C-terminal (CT) cap, usually rich in cysteines, to tie the loose ends of the leucine-rich repeats. By "LRRNT2", the authors mean the leucine-rich repeat domain 2 (all Slits have four LRR domains, so this is the second), and not the NT region/cap within this domain. I would remove the "NT" part, and use LRR Domain 2, LRR D2, LRR 2 or something similar.

* While the issue with low ipTM score for the "four-way" multimer remains, the authors have significantly improved their description and report the model as "low confidence". A less objectionable way could be to manually assemble their high-confidence binary complexes into a large complex rather than reporting a low-confidence four-way multimer, but the outcome would likely be very similar, so it's not worth it.

* As it is difficult to interpret and use the multimeric model the authors report in Fig. 5D, the authors/journal should provide the model (and the other AF models) as a supplemental data file in the form of coordinates (e.g., .pdb or .cif files), which would be ≤1 MB each. Otherwise, it is nearly impossible to test and confirm (or invalidate) their models via site-directed mutagenesis of the proposed interface.

Reviewer #3: The authors have satisfactorily addressed most issues raised, including adding precisions and comments in the text for genetic interactions and AlphaFold predictions, correcting reference citations, and having added Table footnotes for statistical analysis of comparisons. The text flow is much improved.

However, providing exact P-Values for Tables comparisons should be included in an addendum or simple Excel file containing all statistical comparisons so that it is clear what comparisons are being made, as offered by the authors. Key results could be bolded on Excel files.

There are typos in new written sections. For example:

- l. 862 states sax-3(ky152), when it should be zy152

- In Abstract, l.62, 'the’ novel idea (not ‘that’)

**********

**Have all data underlying the figures and results presented in the manuscript been provided?**

Large-scale datasets should be made available via a public repository as described in the *PLOS Genetics*  data availability policy, and numerical data that underlies graphs or summary statistics should be provided in spreadsheet form as supporting information.

Reviewer #1: None

Reviewer #2: **No:** Please add/deposit the predicted AlphaFold models as supplemental data (coordinate files).

Reviewer #3: **No:**

**********

PLOS authors have the option to publish the peer review history of their article (what does this mean?). If published, this will include your full peer review and any attached files.

If you choose “no”, your identity will remain anonymous but your review may still be made public.

**Do you want your identity to be public for this peer review?**  For information about this choice, including consent withdrawal, please see our Privacy Policy.

Reviewer #1: No

Reviewer #2: No

Reviewer #3: No

[NOTE: If reviewer comments were submitted as an attachment file, they will be attached to this email and accessible via the submission site. Please log into your account, locate the manuscript record, and check for the action link "View Attachments". If this link does not appear, there are no attachment files.]

**Figure resubmission:**

**Reproducibility:**

To enhance the reproducibility of your results, we recommend that authors deposit laboratory protocols in protocols.io, where a protocol can be assigned its own identifier (DOI) such that it can be cited independently in the future. Additionally, PLOS ONE offers an option to publish peer-reviewed clinical study protocols. Read more information on sharing protocols at https://plos.org/protocols?utm_medium=editorial-email&utm_source=authorletters&utm_campaign=protocols

# Author comment: Author response to Decision Letter 2

**DOI:** 10.1371/journal.pgen.1012303.r004

24 Aug 2026

Attachment Submitted filename: Response to Reviewers.docx

# Editor report: Decision Letter 2

**DOI:** 10.1371/journal.pgen.1012303.r005

**Authors:** Giovanni Bosco, Jeffrey Essner

25 Aug 2026

Dear Dr Lundquist,

We are pleased to inform you that your manuscript entitled "EFN-4/Ephrin converges with SAX-3/Robo, UNC-6/Netrin, and Heparan Sulfate Proteoglycan signaling to control MAB-5/Hox-dependent posterior Q neuroblast migration in Caenorhabditis elegans" has been editorially accepted for publication in PLOS Genetics. Congratulations!

Before your submission can be formally accepted and sent to production you will need to complete our formatting changes, which you will receive in a follow up email. Please be aware that it may take several days for you to receive this email; during this time no action is required by you. Please note: the accept date on your published article will reflect the date of this provisional acceptance, but your manuscript will not be scheduled for publication until the required changes have been made.

Once your paper is formally accepted, an uncorrected proof of your manuscript will be published online ahead of the final version, unless you’ve already opted out via the online submission form. If, for any reason, you do not want an earlier version of your manuscript published online or are unsure if you have already indicated as such, please let the journal staff know immediately at plosgenetics@plos.org.

In the meantime, please log into Editorial Manager at https://www.editorialmanager.com/pgenetics/, click the "Update My Information" link at the top of the page, and update your user information to ensure an efficient production and billing process. Note that PLOS requires an ORCID iD for all corresponding authors. Therefore, please ensure that you have an ORCID iD and that it is validated in Editorial Manager. To do this, go to ‘Update my Information’ (in the upper left-hand corner of the main menu), and click on the Fetch/Validate link next to the ORCID field.  This will take you to the ORCID site and allow you to create a new iD or authenticate a pre-existing iD in Editorial Manager.

If you have a press-related query, or would like to know about making your underlying data available (as you will be aware, this is required for publication), please see the end of this email. If your institution or institutions have a press office, please notify them about your upcoming article at this point, to enable them to help maximise its impact. Inform journal staff as soon as possible if you are preparing a press release for your article and need a publication date.

Thank you again for supporting open-access publishing; we are looking forward to publishing your work in PLOS Genetics!

Yours sincerely,

Jeffrey J Essner

Academic Editor

PLOS Genetics

Giovanni Bosco

Section Editor

PLOS Genetics

Aimée Dudley

Editor-in-Chief

PLOS Genetics

Anne Goriely

Editor-in-Chief

PLOS Genetics

www.plosgenetics.org

BlueSky: @plos.bsky.social

----------------------------------------------------

Comments from the reviewers (if applicable):

----------------------------------------------------

**Data Deposition**

If you have submitted a Research Article or Front Matter that has associated data that are not suitable for deposition in a subject-specific public repository (such as GenBank or ArrayExpress), one way to make that data available is to deposit it in the Dryad Digital Repository. As you may recall, we ask all authors to agree to make data available; this is one way to achieve that. A full list of recommended repositories can be found on our website.

The following link will take you to the Dryad record for your article, so you won't have to re‐enter its bibliographic information, and can upload your files directly:

http://datadryad.org/submit?journalID=pgenetics&manu=PGENETICS-D-26-00323R2

More information about depositing data in Dryad is available at http://www.datadryad.org/depositing. If you experience any difficulties in submitting your data, please contact help@datadryad.org for support.

Additionally, please be aware that our data availability policy requires that all numerical data underlying display items are included with the submission, and you will need to provide this before we can formally accept your manuscript, if not already present.

----------------------------------------------------

**Press Queries**

If you or your institution will be preparing press materials for this manuscript, or if you need to know your paper's publication date for media purposes, please inform the journal staff as soon as possible so that your submission can be scheduled accordingly. Your manuscript will remain under a strict press embargo until the publication date and time. This means an early version of your manuscript will not be published ahead of your final version. PLOS Genetics may also choose to issue a press release for your article. If there's anything the journal should know or you'd like more information, please get in touch via plosgenetics@plos.org.

# Editor report: Acceptance letter

**DOI:** 10.1371/journal.pgen.1012303.r006

**Authors:** Giovanni Bosco, Jeffrey Essner

PGENETICS-D-26-00323R2

EFN-4/Ephrin converges with SAX-3/Robo, UNC-6/Netrin, and Heparan Sulfate Proteoglycan signaling to control MAB-5/Hox-dependent posterior Q neuroblast migration in Caenorhabditis elegans

Dear Dr Lundquist,

We are pleased to inform you that your manuscript entitled "EFN-4/Ephrin converges with SAX-3/Robo, UNC-6/Netrin, and Heparan Sulfate Proteoglycan signaling to control MAB-5/Hox-dependent posterior Q neuroblast migration in Caenorhabditis elegans" has been formally accepted for publication in PLOS Genetics! Your manuscript is now with our production department and you will be notified of the publication date in due course.

The corresponding author will soon be receiving a typeset proof for review, to ensure errors have not been introduced during production. Please review the PDF proof of your manuscript carefully, as this is the last chance to correct any errors. Please note that major changes, or those which affect the scientific understanding of the work, will likely cause delays to the publication date of your manuscript.

Soon after your final files are uploaded, unless you have opted out or your manuscript is a front-matter piece, the early version of your manuscript will be published online. The date of the early version will be your article's publication date. The final article will be published to the same URL, and all versions of the paper will be accessible to readers.

For Research Articles, you will receive an invoice from PLOS for your publication fee after your manuscript has reached the completed accept phase. If you receive an email requesting payment before acceptance or for any other service, this may be a phishing scheme. Learn how to identify phishing emails and protect your accounts at https://explore.plos.org/phishing.

Thank you again for supporting PLOS Genetics and open-access publishing. We are looking forward to publishing your work!

With kind regards,

Sharmila Kamatchi

PLOS Genetics

On behalf of:

The PLOS Genetics Team

Carlyle House, Carlyle Road, Cambridge CB4 3DN | United Kingdom

plosgenetics@plos.org | +44 (0) 1223-442823

plosgenetics.org | Twitter: @PLOSGenetics
